# CGS-BR: Construction and Benchmarking of a Respiratory Behavior Dataset for the Chinese Giant Salamander

**DOI:** 10.3390/ani16081272

**Published:** 2026-04-21

**Authors:** Dingwei Mao, Yan Zhou, Maochun Wang, Chenyang Shi, Yuanqiong Chen, Qinghua Luo

**Affiliations:** 1 School of Computer Science and Engineering, Jishou University, Jishou 416000, China; amao0407@163.com (D.M.); 18390760534@163.com (M.W.); scy202604@163.com (C.S.); 2 College of Life Sciences, Jishou University, Jishou 416000, China; toutou070215@163.com; 3 Hunan Engineering Technology Research Center for Amphibian and Reptile Resource Protection and Product Processing, College of Biological and Chemical Engineering, Changsha University, Changsha 410017, China

**Keywords:** Chinese giant salamander, respiratory behavior, visual dataset, object detection

## Abstract

The Chinese giant salamander (*Andrias davidianus*) is a nationally protected species in China, and its respiratory behavior serves as a key indicator of its physiological state, health status, and biological rhythm. However, research on intelligent monitoring of its respiratory behavior remains limited due to several challenges, including the species’ nocturnal habits, resulting in low image contrast and poor quality in dark environments; extremely subtle breathing movements; and high-cost manual annotation, leading to a scarcity of high-quality annotated visual data. These factors severely constrain the application of deep learning techniques in this field. To support research on respiratory behavior monitoring in the Chinese giant salamander, this study constructs and releases the CGS-BR dataset, which is the first vision-based dataset dedicated specifically to respiratory behavior detection in this species. The dataset was collected under controlled simulated breeding conditions and consists of 1732 images extracted from 215 high-definition video clips. Following a standardized procedure, each complete respiratory cycle is manually annotated into four stages: head-up, diving, exhalation, and inhalation. To validate the effectiveness of this dataset, this study selects YOLOv8n as the baseline model, which balances detection accuracy, speed, and parameter count, enabling efficient giant salamander respiratory detection under limited resources. By comparing it with several representative models, we provide a reliable evaluation of the dataset’s applicability. CGS-BR aims to provide fundamental data support for research on respiratory monitoring in the Chinese giant salamander, laying the foundation for subsequent applications in conservation management, captive breeding, health monitoring, and early disease warning.

## Simple Summary

This study constructs and releases CGS-BR, the first vision-based dataset dedicated to respiratory behavior detection in the Chinese giant salamander. The dataset comprises 1732 manually annotated images covering four respiratory stages: head-up, diving, exhalation, and inhalation. Using YOLOv8n as a baseline, this study validates the effectiveness of the dataset through comparative experiments with several representative object detection models. CGS-BR aims to provide fundamental data support for research on respiratory monitoring, conservation management, and health assessment of this nationally protected species.

## 1. Introduction

The Chinese giant salamander (*Andrias davidianus*) belongs to the class Amphibia, order Caudata, family Cryptobranchidae, and genus *Andrias* [1], and is a Chinese endemic species and the largest extant amphibian in the world [2]. The giant salamander possesses high nutritional value and shows promising potential for development and utilization in the fields of food, health care, and pharmaceuticals [3,4]. Wild giant salamanders are listed as a Class II National Protected Animal in China and are classified as Critically Endangered on the IUCN Red List of Threatened Species [5]. Since the 1950s, the population size and distribution range of the giant salamander have declined dramatically due to habitat loss and fragmentation caused by human activities. To conserve this species, China has established 14 nature reserves since the 1980s, covering a total area of over 355,000 hectares [6]. In recent years, with the continuous maturation of artificial breeding technologies and the expansion of aquaculture scale, the giant salamander has gradually become an emerging farmed species with market potential; however, disease prevention and control during farming remain key bottlenecks constraining the sustainable development of the industry [7].

Respiratory behavior is a core life activity essential for gas exchange and physiological homeostasis. It not only reflects basal metabolic levels but also responds sensitively to environmental changes and individual health status. Therefore, the normality of respiratory behavior is of great significance for health assessment and disease prevention in the Chinese giant salamander. However, systematic research on its respiratory behavior has been relatively limited due to the species’ nocturnal and cryptic nature, as well as the constraints of traditional monitoring methods. Early studies largely relied on manual observation. Song et al. [8] first described the ventilation process of the salamander; Zheng et al. [9] and Chen et al. [10] respectively found that daytime ventilation frequency under outdoor and indoor aquaculture conditions was significantly positively correlated with water temperature. However, these studies primarily focused on pulmonary respiration during the daytime and did not cover the nocturnal active period. It was not until Tian et al. [11] systematically monitored the salamander throughout the day that two distinct patterns—short breathing and long breathing—were identified in pre-spawning individuals, exhibiting a circadian rhythm characterized by “nocturnal activity and diurnal reduction.” This work preliminarily filled the gap in understanding the species’ diel respiratory behavior. Nevertheless, related detection still relies mainly on continuous manual observation, which is inefficient and subjective, making it difficult to meet the demands of large-scale and precise monitoring.

With the advancement of artificial intelligence technology, object detection tasks in computer vision have shown great potential in aquatic organism detection, with most research focusing on underwater fish detection and behavioral analysis. In aquatic animal behavior monitoring, object detection has become a core enabler of automated observation. Deep learning-based object detection algorithms—such as YOLO and its variants, Faster R-CNN, and SSD—have been widely applied to individual localization and behavior recognition in various aquatic species. Recent studies highlight the versatility and dominance of YOLO-based architectures in underwater fish detection; for example, Wang et al. [12] addressed the challenge of early disease detection in intensive aquaculture by proposing an enhanced YOLOv5 network integrated with a context-aware module, achieving high-precision detection of diseased fish under complex lighting and occlusion conditions. Similarly, Wang et al. [13] combined an improved YOLOv5 with the SiamRPN++ tracker for real-time detection and continuous tracking of abnormal fish behavior, significantly enhancing stability and response speed in dynamic scenarios. For general farmed fish detection, Tu et al. [14] proposed a YOLOv8 variant incorporating a channel non-degradation mechanism and spatial cooperative attention, improving accuracy and robustness in low-contrast, high-noise underwater environments. Abdullah et al. [15] introduced YOLO-Fish, a robust model specifically designed for diverse and challenging underwater scenes, achieving superior performance across multiple datasets and further validating the adaptability of the YOLO framework. Moreover, Ouis et al. [16] evaluated various YOLO-based architectures under different turbidity and illumination conditions, demonstrating their reliability in ecological monitoring applications. SSD and its lightweight variants have also shown strong performance in underwater scenarios. Hung et al. [17] employed SSD-MobileNet for multi-species fish classification, while Tian et al. [18] proposed an improved SSD to detect fish exhibiting “belly-up” symptoms. Additionally, Yu et al. [19] developed an adaptive dead-fish detection method using SSD-MobileNet and validated its practicality on resource-constrained edge devices. Applications of Faster R-CNN have extended beyond fish detection. As early as 2015, Li et al. [20] pioneered its use for underwater fish detection. Subsequently, Zeng et al. [21] enhanced its robustness under severe occlusion by introducing an adversarial occlusion network. Notably, Gu et al. [22] utilized an improved Faster R-CNN to achieve automatic sex identification of juvenile Chinese mitten crabs, overcoming the low efficiency and high error rates of manual sorting and enabling better seedling management. Furthermore, Moniruzzaman et al. [23] applied Faster R-CNN to detect seagrass from underwater digital images, demonstrating its effectiveness in benthic habitat monitoring—a critical component of marine ecosystem assessment. Finally, Fan et al. [24] explored AI-driven behavior recognition in zebrafish by constructing a deep learning-based classification framework, offering a new approach for high-throughput phenotypic analysis in this model organism.

However, despite the remarkable achievements of AI-driven object detection in monitoring various aquatic species such as fish, its application in the behavioral ecology of the Chinese giant salamander remains virtually unexplored. Currently, observation of salamander respiratory behavior primarily relies on manual video playback and counting—a time-consuming and labor-intensive process that is prone to subjective bias and incapable of enabling long-term, high-frequency continuous monitoring. This limitation is particularly pronounced for a species with strong cryptic habits that frequently engages in nocturnal or cave-dwelling activities, where traditional methods suffer from significant deficiencies in data completeness and objectivity. Notably, apart from our team’s recent study by Li et al. [25] on parental care behavior recognition in giant salamanders using an improved YOLOv8 model, no publicly available research has yet applied YOLO or other mainstream deep learning detection frameworks for the automatic identification of individual salamanders or the precise capture of their respiratory behaviors. This research gap is largely attributable to the scarcity of annotated behavioral datasets for the giant salamander, which severely constrains the potential of artificial intelligence in its conservation.

Artificial intelligence-based monitoring systems have become increasingly mature, with their application value extending beyond the systems themselves toward the development of secure, intelligent, data-driven monitoring ecosystems [26,27]. Against this backdrop, constructing a high-quality, finely annotated visual dataset is of critical importance for overcoming the current bottlenecks in accuracy and reliability of intelligent respiratory behavior monitoring in the Chinese giant salamander, filling the data gap in this field, and unlocking the potential of artificial intelligence applications. To this end, this study presents the first release of CGS-BR, a dataset dedicated specifically to respiratory behavior detection in the Chinese giant salamander, aiming to provide a reliable data foundation for deep learning-based object detection and behavioral analysis, thereby supporting AI-driven species conservation and health assessment. In addition, this study validates the effectiveness of the dataset through benchmark experiments, with the goal of providing a reference baseline for subsequent research.

## 2. Materials and Methods

### 2.1. Source of Materials

**Figure 1:**
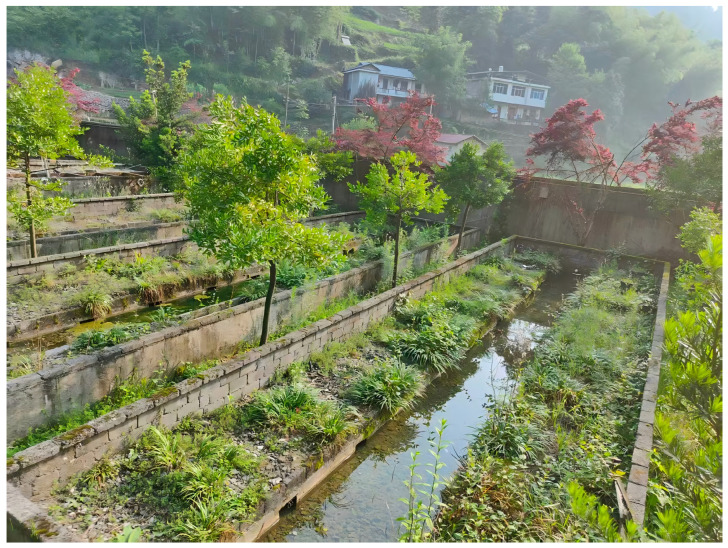
Schematic diagram of the simulated ecological environment for the Chinese giant salamander.

The experimental data used in this study were collected at the ecological breeding facility of Zhuyuan Giant Salamander Biotechnology Co., Ltd., located in Kongke Shu Township, Sangzhi County, Zhangjiajie City, Hunan Province, China (geographic coordinates: 29°28′ N, 110°22′ E; elevation: 471 m). The facility features an artificial breeding system designed to mimic the natural habitat of the Chinese giant salamander (*Andrias davidianus*) to support research on its reproductive behavior and conservation efforts, as illustrated in Figure 1.

The breeding pond contains an artificial stream approximately 22 m in length, with an average width of 1.10 m and a water depth of about 0.40 m. It is equipped with three independent cave units. Each cave has an internal floor area of approximately 1.44 m^2^, with dimensions of roughly 2.40 m in length, 0.30 m in width, and 0.25 m in height, and a substrate depth of approximately 0.20 m. The interior of each cave is maintained under continuously low illumination, presenting a dark environment consistent with natural caves. The cave floors are covered with natural sand, gravel, and cobblestones to replicate the microhabitat characteristics preferred by wild giant salamanders, which typically shelter in rock crevices or burrows. Each cave houses one female and one male giant salamander, both wild F2 generation individuals, with body lengths ranging from 1.12 m to 1.26 m, body weights from 9.80 kg to 12.20 kg, and ages from 9 to 10 years. All experimental subjects were reared under controlled simulated aquaculture conditions to retain the genetic and behavioral traits representative of wild populations.

**Figure 2:**
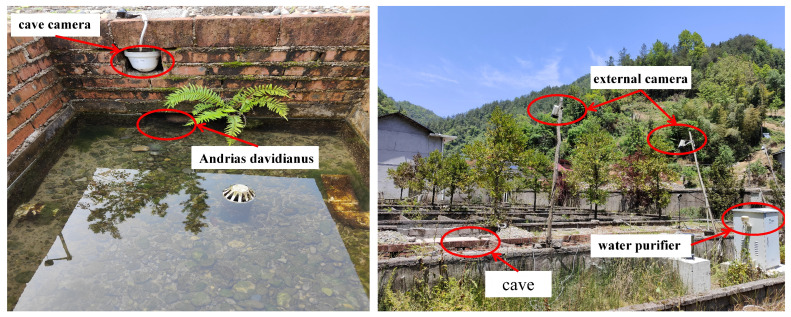
Camera setup for cave monitoring.

To comprehensively capture behavioral dynamics both inside and outside the caves—particularly key metrics such as respiratory frequency, activity rhythms, and social interactions during the breeding season—we installed high-definition infrared network cameras (model: HIKvision DS-2CD3T35D-I5; Hangzhou Hikvision Digital Technology Co., Ltd., Hangzhou, China) at the observation window and directly above each cave, as shown in Figure 2. Video acquisition was configured with a resolution of 2560 × 1040 pixels, a frame rate of 50 fps, and HEVC/H.265 video encoding to balance image fidelity with storage efficiency.

Notably, despite the vast volume of raw video data, the slow movement and low breathing frequency of giant salamanders, resulting in subtle transitions between consecutive behaviors, posed significant challenges during the annotation process, which is the primary reason for the relatively limited size of the final dataset. Data collection spanned a full annual cycle, from 1 December 2022 to 30 November 2023. Over this period, approximately 8000 h of raw video footage were recorded, amounting to over 40 TB of data. Following an initial quality screening process—which excluded clips affected by equipment failure, lens obstruction, or severe blurring—we extracted non-duplicate, clear key frames to construct the Chinese giant salamander breathing behavior dataset, designated as CGS-BR. This dataset consists of high-resolution images that have been meticulously and expertly annotated.

### 2.2. Dataset Construction

The dataset constructed in this study is primarily designed for object detection tasks, aiming to accurately localize key behavioral postures of the Chinese giant salamander during pulmonary respiration. Object detection is a fundamental task in computer vision, with the core objective of automatically identifying objects of specific categories in images and delineating their positions and extents using bounding boxes. In this study, the target of interest is individual giant salamanders at different respiratory stages.

**Figure 3:**
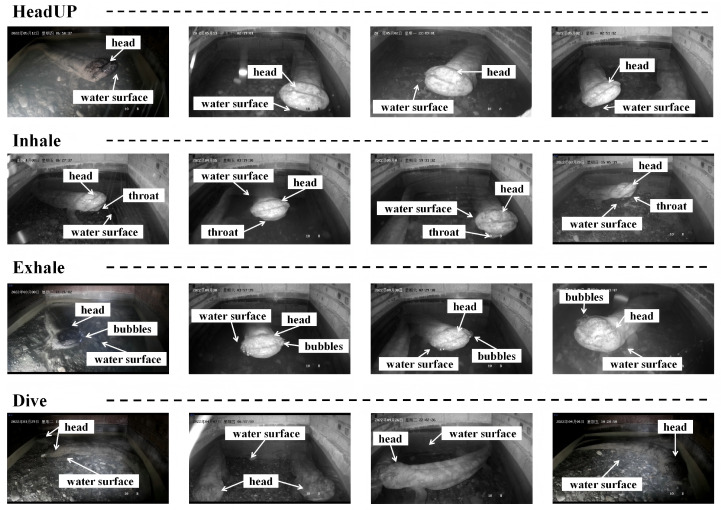
Schematic of behavioral classification in the Chinese giant salamander.

The CGS-BR dataset follows the classical definition of pulmonary respiration behavior in Chinese giant salamanders proposed by Song et al. [8] and Zheng et al. [9]: “A complete pulmonary respiration cycle is defined as the animal raising its head above the water surface to inhale, followed by submerging its head underwater to exhale.” Based on this definition, we further decompose a complete respiration cycle into four consecutive action phases: HeadUP, Dive, Exhale, and Inhale, as illustrated in Table 1 and Figure 3. To avoid redundant sampling caused by high inter-frame similarity in video sequences, we carefully selected and extracted 1732 representative image frames from 215 high-quality video clips, ensuring that each frame has sufficient diversity and independence in terms of behavioral phase, viewing angle, and body posture. This process was achieved by performing manual annotation only when the giant salamander exhibited obvious movement behaviors, so as to minimize the occurrence of similar duplicate frames in the dataset. In terms of data partitioning, we adopted a video-level stratified principle: each video clip recording a complete respiratory behavior is taken as the basic unit, and all image frames extracted from the same clip are collectively assigned to one of the training, validation, or test sets. This prevents highly similar consecutive frames from appearing across different subsets, thereby avoiding data leakage and falsely inflated model performance.

**Figure 4:**
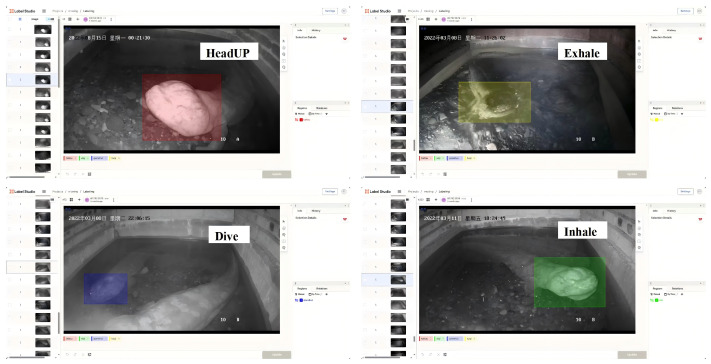
Label Studio labeling interface.

During the dataset annotation phase, we used the open-source data labeling platform Label Studio (https://labelstud.io/) to annotate bounding boxes around the Chinese giant salamander in each image and assign the corresponding respiratory behavior category, as shown in Figure 4.

**Figure 5:**
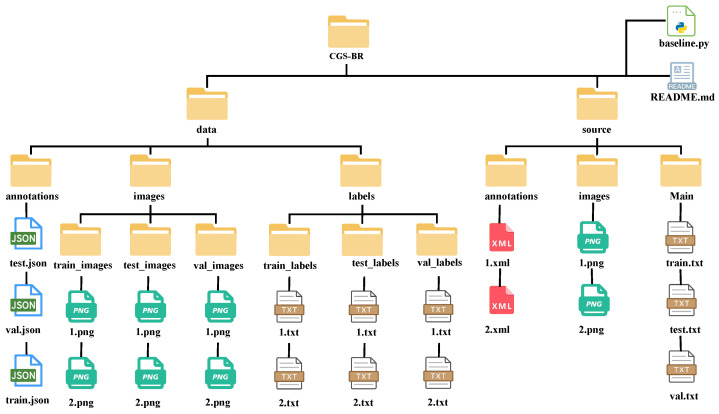
Directory organization and file composition of the CGS-BR.

The CGS-BR dataset adopts a modular hierarchical structure, with its root directory primarily containing two core data folders, data and source, along with a README.md documentation file and a baseline.py script. The data directory is specifically designed for deep learning training and is subdivided into three folders: annotations, which stores COCO-format train.json, val.json, and test.json files; images, which contains standardized PNG images categorized into train_images, val_images, and test_images; and labels, which provides YOLO-format train_labels, val_labels, and test_labels text files containing normalized coordinates, thereby enabling immediate compatibility with multiple annotation formats. In contrast, the source directory preserves raw materials and underlying resources, including PASCAL VOC-standard XML annotation files (e.g., 1.xml), unprocessed high-resolution original PNG images (e.g., 1.png), and index lists (train.txt, val.txt, and test.txt) located within the Main subfolder to explicitly define dataset splits and support user-defined data cleaning or re-annotation workflows. This dual-track design, combining standardized ready-to-use data with raw sources, not only facilitates rapid model reproduction but also provides comprehensive support for in-depth data tracing and extended research, as illustrated in Figure 5.

## 3. Baseline Model

### 3.1. Model Selection

To concretely evaluate the applicability and challenges of the CGS-BR dataset in practical object detection tasks, we selected YOLOv8n as our baseline model. Considering that respiratory behavior recognition in Chinese giant salamanders may be applied in field monitoring systems or long-duration video analysis scenarios, factors such as inference efficiency, model compactness, and deployment simplicity are of practical relevance. Therefore, this study adopts YOLOv8n, the smallest variant in the YOLOv8 family, as the primary baseline model.

YOLOv8n was released by Ultralytics [28] as the latest iteration of the YOLO series. It significantly improves detection accuracy and training stability while maintaining real-time inference capability. Key advantages include: (1) an anchor-free detection head that simplifies label assignment and enhances sensitivity to small objects; (2) the introduction of the C2f module in the backbone network, which employs denser cross-stage connections and feature reuse mechanisms to improve feature representation capacity and gradient propagation efficiency; (3) a refined loss function that integrates distribution focal loss with task-aligned learning, achieving greater consistency in positive–negative sample matching and bounding box regression; and (4) a complete model scaling spectrum ranging from the lightweight YOLOv8n to the high-performance YOLOv8x, enabling flexible deployment under varying computational constraints. Furthermore, the framework provides a highly integrated toolkit for training, validation, inference, and model export, supporting one-click conversion to multiple formats such as ONNX and TensorRT. These features collectively lower the engineering barrier for real-world deployment and make YOLOv8n particularly well-suited for field-based video surveillance scenarios that require robust performance under limited computational resources.

### 3.2. Experimental Setup

All experiments were conducted on a cloud server equipped with an NVIDIA RTX 4090D GPU (24 GB VRAM) and 80 GB of system memory. The software environment consisted of Ubuntu 20.04, CUDA 12.1, Python 3.12, and PyTorch 2.3.0. Detailed specifications of the experimental setup are provided in Table 2. Input images were uniformly resized to 640 × 640 pixels, with a batch size of 32 and a total of 30 training epochs.

### 3.3. Evaluation Metrics

To objectively evaluate the performance of the proposed method, this study adopts a comprehensive set of evaluation metrics—including average precision (AP) and mean average precision (mAP)—which are used to assess the model’s accuracy. These metrics collectively reflect the model’s scale, computational complexity, and detection efficiency.

TP denotes the number of target frames correctly predicted as positive instances, FP represents the number of target frames incorrectly classified as positive (false alarms), and FN denotes the number of positive instances that are misclassified as negative (missed detections).

*Precision* is the ratio of true positive detections to all positive predictions made by the model, defined as

(1) $$\mathrm{Precision}=\frac{TP}{TP+FP}$$

*Recall* is the ratio of the number of targets correctly predicted as positive instances to the total number of actual positive instances, defined as

(2) $$\mathrm{Recall}=\frac{TP}{TP+FN}$$

*AP* is the area under the Precision-Recall curve, representing the average precision of the model across different recall levels, defined as

(3) $$\mathrm{AP}=\int_{0}^{1}\mathrm{Precision}\;d\left(\mathrm{Recall}\right)$$

*mAP* is a comprehensive metric used to evaluate the performance of object detection models across multiple categories. It computes the Average Precision (AP) for each class and then takes the mean of these AP values to provide an overall measure of model performance, as follows:

(4) $$\mathrm{mAP}=\frac{\sum_{i=1}^{N}\mathrm{AP}\left(i\right)}{N}$$

where *N* denotes the number of classes in the dataset (in this study, N=4). A higher mAP value indicates better model performance. *Precision* and *Recall* are dimensionless metrics, typically expressed as percentages, representing the ratio of correctly predicted instances to the total number of predictions.

Additionally, in the experiments, we first performed a stratified split on the CGS-BR dataset based on the distribution proportions of the four respiratory behavior categories (Dive, HeadUP, Exhale, Inhale), to ensure that the training set, validation set, and test set each retain a class distribution similar to that of the original dataset. On this basis, we allocated 1272 images to the training set, 240 images to the validation set, and the remaining 220 images to the test set out of the total 1732 images in the CGS-BR dataset, following an approximate ratio of 7.3:1.4:1.3, thereby ensuring effective model training, feasible hyperparameter tuning, and reliable evaluation results, as shown in Table 3.

## 4. Experimental Results

**Figure 6:**
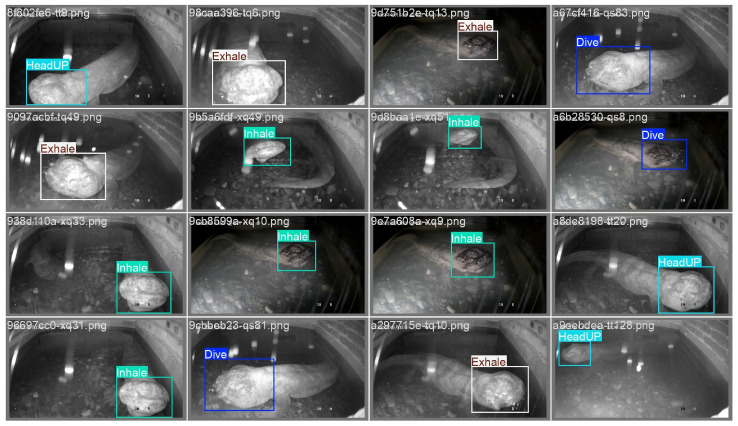
Baseline model recognition performance on the dataset.

**Figure 7:**
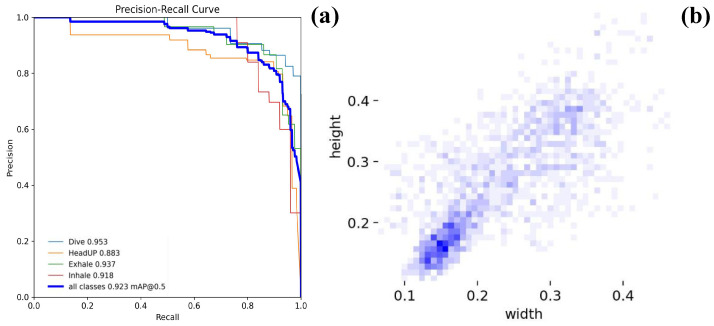
(**a**) Precision–Recall curves; (**b**) Bounding box height-width distribution.

We trained and evaluated the YOLOv8n model on image data capturing the respiration behavior of Chinese giant salamanders (CGS) in a simulated aquaculture pond environment. Figure 6 presents the detection results of YOLOv8n on one test subset of the CGS-BR dataset, demonstrating that the model accurately identifies respiration actions of the salamanders. The model achieved a performance of mAP@0.5 = 92.3% on the test set, with average precision (AP) for individual respiration behaviors all exceeding 0.88, as shown in Figure 7a.

We emphasize that this result is not intended to highlight methodological novelty, but rather to establish an open and reproducible benchmark for CGS-BR, enabling future researchers to evaluate new algorithms under consistent conditions. As the first vision-based dataset dedicated specifically to respiration behavior detection in Chinese giant salamanders, CGS-BR fills a critical data gap in behavioral monitoring of this species, and holds promise for supporting both captive breeding management and wild conservation efforts.

As shown in Figure 7b, the bounding boxes corresponding to respiration behaviors generally exhibit small scales and near-rectangular shapes, with aspect ratios concentrated in the range of 0.1–0.4. This indicates that such actions possess stable spatial structural characteristics. The consistency in both semantic annotation and geometric layout demonstrates that CGS-BR exhibits high regularity not only in labeling but also in spatial configuration, thereby providing a reliable data foundation for high-precision detection.

**Figure 8:**
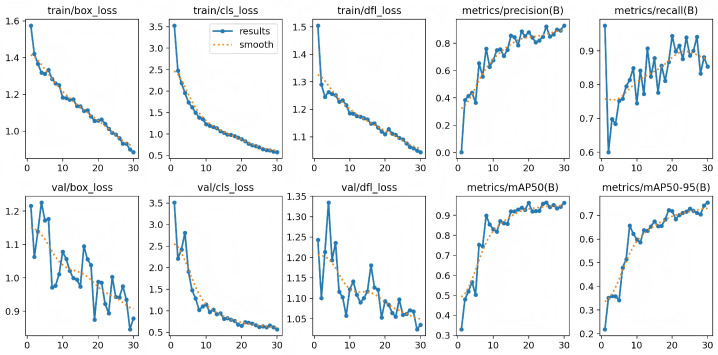
Training curves of the baseline model over 30 epochs.

Figure 8 illustrates the training process of YOLOv8n on our constructed CGS-BR dataset. As the number of training epochs increases, all loss components steadily decrease, while validation metrics—including precision, recall, and mAP—consistently improve, ultimately achieving a highly satisfactory performance. This result demonstrates that our dataset is capable of effectively supporting the training of a standard object detection model. In other words, CGS-BR is a practical, trainable, and results-producing resource that lays a reliable foundation for future automated analysis of Chinese giant salamander respiration behavior.

**Figure 9:**
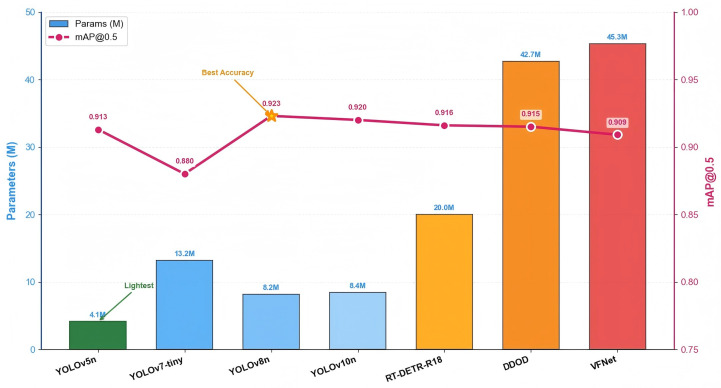
Comparison of parameters and detection accuracy across different object detection models.

In addition, to further analyze the performance of other mainstream models on the giant salamander respiratory behavior dataset, we conducted a comparative experiment on seven typical object detection models—namely, YOLOv5n, YOLOv7-tiny, YOLOv8n, YOLOv10n, DDOD, VFNet, and RT-DETR-R18—using AP (Dive, HeadUP, Exhale, Inhale), mAP@0.5, mAP@0.5:0.95, Params (M), GFLOPs, and FPS as the evaluation metrics, as shown in Table 4 and Figure 9. Overall, all the above mainstream object detection models achieved satisfactory performance on the giant salamander respiratory behavior dataset, which to some extent validates the effectiveness and reliability of this dataset. However, it should be noted that this conclusion is limited to the controlled environment of this study, and the generalization capability of this dataset in broader scenarios remains to be further examined.

## 5. Discussion

The CGS-BR dataset addresses the inherent challenges in respiratory behavior recognition of the Chinese giant salamander, such as subtle motion amplitudes and low imaging contrast, by employing targeted acquisition strategies including high-frame-rate video capture, multi-angle illumination control, and optimized camera deployment in standardized aquaculture ponds. The benchmark experimental results demonstrate that these measures effectively support the construction of a usable dataset, and lightweight models achieve reasonable detection performance on this dataset.

Although the dataset performs well in standardized aquaculture environments, the current version still has certain limitations. First, the data acquisition scenario is primarily a simulated aquaculture environment, which differs from unstructured wild habitats in terms of background complexity and individual morphological variation, requiring further adaptation for direct application to wild scenarios. Second, the experimental results based on existing mainstream object detection models reveal performance variations across different respiratory behavior categories, with detection accuracy under low-light nighttime conditions still having room for improvement.

To address the above limitations, future work will focus on two aspects: data and algorithms. On the data side, we plan to further expand the CGS-BR dataset to include individual data from different age groups, different body weights, and different health statuses, while covering more diverse environments and scenarios, evolving it into a continuously updated open resource rather than a static benchmark dataset. On the algorithm side, we will explore more advanced model architectures and training strategies, with a focus on improving the accuracy and robustness of nighttime behavior detection. Through the iterative synergy of data and technology, we aim to advance the development of automated monitoring tools for the Chinese giant salamander, providing more reliable technical support for its conservation practices.

## 6. Conclusions

In conclusion, this paper introduces CGS-BR, a visual dataset for respiratory behavior detection in the Chinese giant salamander. The dataset comprises 1732 manually annotated images covering four phases of the respiratory cycle, collected under controlled aquaculture conditions with targeted strategies to address challenges such as subtle motion and low contrast. Benchmark experiments demonstrate that CGS-BR provides a viable foundation for training detection models. While the dataset was collected in a controlled aquaculture environment, which differs significantly from complex wild cave environments characterized by variable lighting, water flow variation, and occlusions, posing challenges for its direct application, we still regard the dataset as an initial step toward intelligent monitoring of this species. Future work will focus on two aspects: on the data side, we plan to expand the dataset to bridge the gap between controlled and real-world environments; on the algorithm side, we will explore more advanced model architectures and training strategies, with a focus on improving the accuracy and robustness of nighttime behavior detection, ultimately supporting conservation efforts for the Chinese giant salamander.

## Figures and Tables

**Table 1:** Definition of lung breathing behavior of the Chinese giant salamander.

Behavior	Definition
HeadUP	*Andrias davidianus* raises its head above water
Inhale	*Andrias davidianus* opens mouth and pharynx, inhales air
Dive	*Andrias davidianus* submerges its head under water, nostrils submerged
Exhale	*Andrias davidianus* exhales air from lungs underwater, bubbles visible

**Table 2:** Training environment and hardware parameter platform.

Category	Configuration
CPU	18 vCPU AMD EPYC 9754 128-Core Processor
GPU	RTX 4090D (24 G)
System environment	Ubuntu 20.04
Framework	PyTorch 2.3.0
Programming language	Python 3.12

**Table 3:** CGS-BR dataset statistics.

Class	Train	Val	Test	Total
HeadUP	467	88	85	640
Inhale	203	38	37	278
Dive	267	50	48	365
Exhale	335	64	50	449

**Table 4:** Performance Comparison Across Models on the CGS-BR Dataset, where bold values denote the optimal values under the respective metrics.

Model	Dive	HeadUP	Exhale	Inhale	mAP@0.5:0.95	mAP@0.5	Params (M)	GFLOPs	FPS
DDOD	0.902	0.926	0.912	0.920	0.688	0.915	42.7	32.20	35.2
VFNet	0.908	0.912	0.904	0.913	0.695	0.909	45.3	32.72	32.8
RT-DETR-R18	0.912	0.918	0.914	0.920	**0.721**	0.916	20.0	60.91	45.5
YOLOv5n	0.892	0.928	0.916	0.921	0.690	0.913	**4.1**	**1.76**	**124.5**
YOLOv7-tiny	0.862	0.918	0.882	0.858	0.642	0.880	13.2	6.02	98.3
YOLOv8n	**0.916**	0.886	**0.938**	**0.952**	0.710	**0.923**	8.2	3.01	112.6
YOLOv10n	0.908	**0.932**	0.916	0.924	0.698	0.920	8.4	2.27	108.4

## Data Availability

The CGS-BR dataset is publicly available in the Zenodo repository under the DOI: https://doi.org/10.5281/zenodo.18899820. The dataset involves the Chinese giant salamander (*Andrias davidianus*), a Class II nationally protected animal in China. Given this protected status and associated ethical considerations for species conservation, public access to the dataset files is restricted strictly to non-commercial academic use. Researchers are encouraged to contact the corresponding author to sign a data use agreement and obtain download permissions.
